# Understanding the Washing Damage to Textile ECG Dry Skin Electrodes, Embroidered and Fabric-Based; set up of Equivalent Laboratory Tests

**DOI:** 10.3390/s20051272

**Published:** 2020-02-26

**Authors:** Shahood uz Zaman, Xuyuan Tao, Cédric Cochrane, Vladan Koncar

**Affiliations:** 1 École Nationale Supérieure des Arts et Industries Textiles/Génie et Matériaux Textiles laboratory (ENSAIT/GEMTEX), 2 Allée Louis et Victor Champier, F-59100 Roubaix, France; xuyuan.tao@ensait.fr (X.T.); cedric.cochrane@ensait.fr (C.C.); vladan.koncar@ensait.fr (V.K.); 2 GEMTEX, University of Lille, Cité Scientifique, F-59650 Villeneuve d’Ascq, France

**Keywords:** skin electrode, launderability, washability, E-textile, ECG, wash forces

## Abstract

Reliability and washability are major hurdles facing the e-textile industry nowadays. The main fear behind the product’s rejection is the inability to ensure its projected life span. The durability of e-textiles is based on an approximate lifetime of both the electronics and textiles integrated into the product. A detailed analysis of the wash process and the possibility of predicting product behavior are key factors for new standards implementation. This manuscript is focused on the washability issues of different types of woven, knitted, and embroidered, textile-based ECG electrodes. These electrodes are used without the addition of any ionic gel to the skin to reduce impedance. They were subjected to up to 50 wash cycles with two different types of wash processes, and changes in surface resistance, as well as the quality of ECG waves, were observed To investigate the wash damages in detail, the proposed mechanical (Martindale and Pilling box) and chemical test methods were investigated. The electrodes which increased resistance after washing showed the same trend in the proposed test methods. Copper-based electrodes suffered the most severe damage and increased resistance, as was also visible in an SEM analysis. These proposed test methods can be used to predict robustness behavior without washing.

## 1. Introduction

In the modern era, human habits quite different to those of ancient times. In recent decades, advances have been based on technological developments; scientists have recently developed a number of innovative notions that we couldn’t even have imagined in past years. To address the recent speed of advancement in our daily lives, different fields have merged to yield state-of-the-art products [1]. The most noteworthy examples are the use of electronic products in the textile and automotive industries. Current automotive and aerospace assembly includes a lot of user-friendly gadgets and IoT (Internet of Things) -based technology, including inner light decoration on the dashboard, smart displays, seat heating, etc. [2,3]. Similarly, modern textiles, especially textile wearable products are transformed into wearable electronics. These wearable e-textiles have been developed based on output requirements. They have the ability to sense and respond to the environment or store data for further usage. These products are being widely used in the medical, sports, personal protection, communication, and fashion industries [4,5,6]. 

In the medical industry, wearable e-textiles are being used to monitor physical activities, such as real-time ECG measurements with the help of embedded conductive textile sensors. These innovations are bringing about new possibilities and awareness of the modernized health sector. In the field of personal protection (PPE), different monitoring devices embedded in clothing are available for firefighters and military personnel. These achievements allow the authorities to acquire real-time information regarding hazardous situations or threats faced by their operatives. These innovations have also changed the performance monitoring analysis criteria in sports. Modern technologies are being widely used in footwear to monitor athletes’ running foot balance, in swimsuits for body movement analyses, in equipment such as tennis rackets to calculate potential vibrations, etc. [7,8,9,10,11]. Now, wearable textiles are going beyond mere clothing purposes. Currently, these products are in the transition phase from scientific laboratories to common usage; however, the vast majority of products on the market are still struggling to gain customer confidence. Seamless integration into daily life is required without any discomfort or need to take extra precautions [12,13]. 

The main fear behind the product’s rejection is the inability to ensure its projected life span. The durability of an e-textile is based on achieving a target-based lifetime of both the electronics and textiles integrated together into one product. These products undergo different levels of impacts, based on their usage. The most damaging and destructive forces are related to the washing process. Each wearable e-textile will be washed multiple times during its life span. This includes both textiles and the electronic components integrated therein as a complete unit. At present, researchers are shifting their focus to the reliability and washability of e-textile products [14,15]. 

Some literature is available on the washability of e-textile products. Olivia et al. [15] examined the effect of 20 wash cycles on e-textile sensors. They calculated the proximity and touch detection after the wash cycles. They claimed that the e-textile circuits survived 10–15 wash cycles, but that the proximity detection time increased by up to 145%, and touch detection increased by up to 142%. Microscopic analyses showed that wires were pulled out from solder joints due to mechanical forces including bending and twisting. Molla et al. [14] washed e-textiles with surface-mounted LEDs soldered with stitched traces. They prepared a separate set of samples for stitched traces only and stitched traces soldered with LEDs on them. These samples were protected with different types of materials and underwent up to 10 wash cycles in a household washing machine. They claimed that, in the best case, i.e., stitched traces protection, resistance was increased from 0.24 to 0.58Ω/m. In the case of LEDs soldered with conductive traces, 3–4 LEDs out of 5 were still working after 10 wash cycles. Lee et al. [1] studied the washability of different metal-coated conductive fabrics. These fabrics were sandwich laminated with thermoplastic urethane (TPU) and three different base materials. The electrical resistance in the function of launderability was calculated after 10 wash cycles. Silver-based fabric showed the least change in resistance after 10 wash cycles among all the types of base fabric laminations. They claimed that adhesion between nylon and copper/nickel metal was not good, and that metal peeled out during washing. Berglund et al. [16] washed polymer film insulated and noninsulated body sensors up to 5 times. They claimed that both were affected minimally after machine washing, but they suggested that tumble drying should be avoided. Melkie et al. [17] prepared PEDOT:PSS coated textile fabrics with some additives (i.e., glycol, polyurethane, methanol etc.) on the fabric substrate. They provided a coated polyester fabric with additives immersion instead of coating, and claimed that conductivity enrichment occurred by the formation of hydrogen bond with additives and the removal of PSS. They dipped the samples in liquid additives and claimed that the surface tension, as well as surface resistance, was reduced. These samples underwent up to 10 wash cycles and conductive stability was observed. 

Research dealing with the washability and reliability of different e-textile prototypes is available. However, it has only focused on the washing life of different products. Detailed analyses of the wash process and the ability to predict product behavior still need to be addressed. Well-developed standards are available for both the textile and electronic industries. There is therefore a need to define new reliability standards for this new hybrid industry. A lot of organizations are working on new standards, including AATCC (https://www.aatcc.org), IEC (https://www.iec.ch), and IPC (https://www.ipc.org). IPC (Association connecting electronics industries) is working to define the standard for electronics industries so that they can integrate their products in the e-textile market. For the long-term success of the e-textile market, these standards should be developed quickly to boost market capitalization. This research investigated e-textile washability and the prediction of the life span of e-textile products without actually washing them. A detailed analysis of possible wash forces was conducted in these experiments. These standards will help other industries, especially electronics industries, to develop products which are suitable for textile integration without fear of rejection. In this research, the effect of washing on conductive skin electrodes was observed.

**Figure 1:**
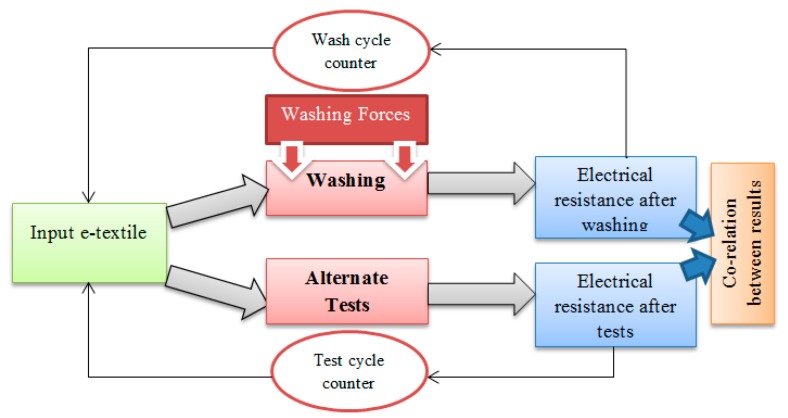
Experimental approach.

Embroidered and fabric-based (knitted and woven) textile ECG dry skin electrodes were prepared. These electrodes underwent up to 50 wash cycles, and changes in their sheet resistance, along with output ECG signals, were measured. Based on the acting forces in the wash process, alternative tests have observed equivalent levels of damage. These tests can be used to predict the washing life of e-textile components without washing. Figure 1 explains the approach of these experiments.

## 2. Materials and Methods

### 2.1. Skin Dry Electrodes

We used fabric-based skin electrodes because they are the most feasible and easily available on the market. These types of electrodes are widely used in experiments nowadays. The idea was to use as many variations as possible in our experiments. However, no linking degradation mechanism is possible among them, because they have different structures. In fact, this degradation coefficient will be different for different types of e textile prototypes, and should be analyzed separately.

Conductive fabric was used to create the ECG skin electrodes. Four different types of conductive fabrics were used in this experiment (F1–F4, as in Table 1). F2 and F3 were metallic polyester fabric coated with 100% copper and copper–nickel, respectively. F1 and F4 were silver-plated, knitted fabrics. F1, F2, and F3 were bought from Faraday Defense LLC, USA. F4 fabric was bought from Innovative textiles, Italy.

In the second part of experiment, two different types of embroidered skin dry electrodes were developed (E1 and E2 in Table 1). These electrodes were used for ECG measurements in real-time, data transmission, and analysis. Embroidered skin electrodes were prepared based on previous work [18]. These electrodes were embroidered using two different types of yarns: Shieldex (117f17 HC+B) (E1) and Madeira (HC-40) yarns (E2). 

Four to six samples for each type of electrode and test were prepared. Different instruments have different sampling capacities. For example, in the case of the abrasion test, five samples can be tested at the same time, but this is limited to four in case of the pilling box test. Similarly, for an embroidery machine, four samples can be prepared together. The maximum capacity for each machine to get a better average and to avoid any repetition during experiments was used. These electrodes were stitched on plain 100 % cotton fabric, and each sample consisted of three electrodes placed at appropriate positions for ECG measurement.

### 2.2. Surface Resistance

The surface resistance of the skin electrodes was measured using a Four-Point Probe system (Ossila, United Kingdom). Four needles with a 0.24 mm radius having 1.27 mm spacing were used. The outer two probes are used to achieve the targeted current; once achieved, voltage is measured via the inner two probes. The source measurement unit (SMU) was used to display the sheet resistance in terms of Ohms per square unit.

### 2.3. Washing

To wash ECG skin electrodes, a domestic washing machine, the MIELE W3240, from France was used. ISO 6330 standards were followed for the washing of these samples. Two different wash programs, “Express” and “Silk”, were used for separate sets of each type of skin electrode. These programs were selected based on previous research conducted on different wash programs and their behavior [8]. A major difference between both processes was the speed of the wash process. The wash speeds were 15 RPM and 38.5 RPM for the silk and express wash cycles, respectively. The tumbling speed in both cases was 400 RPM. Wash time was almost 35 min, and temperature was 40 °C for both processes. X.TRA Total detergent was used in this experiment. For each wash, 20 g of detergent based on ISO 6330 standards (1.25 g/L) was used. The wash load was maintained at 2 kg, as per standards. All samples underwent up to 50 wash cycles.

### 2.4. Martindale Abrasion Test

Abrasion damage was recorded on a Martindale Abrasion tester (James H. Heals & Co Ltd. United Kingdom) using 9 K.Pa load on the samples. Standard woven felt was used on a lower fixed surface. The unidirectional movement of the top plate was used for this experiment. The abrasion test comprised up to 10,000 abrasion cycles. The top plate speed was 0.8 sec/cycle and the total distance covered in each cycle was 5.5 cm.

### 2.5. Pilling Box Test

An orbiter pilling and snagging tester (James H. Heals & Co Ltd., United Kingdom) was used to observe the pilling performance of these skin electrodes. The electrodes were stitched onto plain cotton fabric, and the fabric was shaped into a tube form by stitching both edges together. The pilling box speed was adjusted to 60 RPM, and the testing conditions were set to 20 ± 2 °C and 65 ± 5 % R.H. All samples were tested for 10,000 Pilling cycles.

### 2.6. Water and Detergent Solutions Analysis

During the wash process, certain forces acted on the skin electrodes. Major forces are mechanical and chemical in nature. Water and detergent solutions may react and attack the skin electrodes undergoing the wash process. To investigate the chemical impact separately, these electrodes were immersed in water and detergent solutions for a duration varying from 30 min to 72 h. Five samples for each type of electrode were used. The ratio of detergent:water in solution was maintained at 1–1.25 g/L, as described in the ISO 6330 standards. The temperature of these solutions was maintained at 40 °C, i.e., similar to the wash process temperature.

### 2.7. ECG Measurement

**Figure 2:**
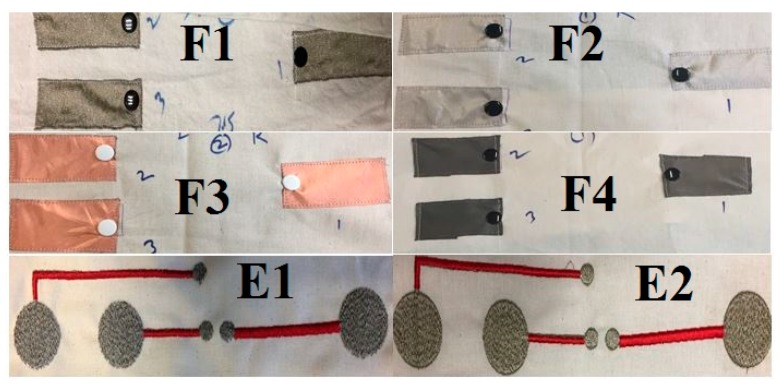
Different types of textile dry skin electrodes.

ECG measurement was performed using the SHIELD-EMK-EMG card from OLIMEX. This card was connected to an Arduino board. Figure 2 shows the ECG electrodes for different types of materials. For fabricated samples (F1–F4), the size of the electrodes was 3 cm × 6 cm. For E1 and E2, circular electrodes with 3 cm diameter were embroidered. The sampling frequency was 250 Hz and the data were processed and analyzed using MATLAB software. A notch filter was used to remove powerline noises at 50 Hz and a passband filter was used to filter 0.5 to 100 Hz. Signals were recorded in still sitting conditions for at least 45 seconds. Before measurement, subjects were in a resting position for at least 10 min. These experiments were conducted on three healthy male subjects aged between 26 to 38 years. It was obvious that factors such as contact pressure, textile placement, and particularly, user activity (sitting, running, laying) strongly influence the recorded ECG signals. In some cases, the numerical filtering (low pass and notch filters) may significantly improve the ECG signals performance. This is true for motion electrodes; when the pressure between the electrodes and skin changes, the electrode-to-skin contact impedance varies strongly and rapidly. This effect may be attenuated by low pass filtering. The influence of a 50 Hz power supply disturbance which is often visible in the ECG signal may be removed using a notch filter. Even if these parameters are very interesting and important, this was not the main objective of our study. This article focuses on the washability factors influencing the functionality and lifetimes of textile ECG dry skin electrodes. Electrocardiographic waves consist of a P wave, T wave, and QRS complex [18,19,20]. If these peaks are visible in the graph, we can state that the ECG signals are being properly detected.

## 3. Results

### 3.1. Washability

In the wash process, different forces act simultaneously on the specimens. These forces impact upon the life span of e-textile products. The major wash factors are mechanical and chemical forces. During washing, fabrics undergo different types of bending, twisting, tangling, and stretching. Similarly, detergent and the water itself can react during the wash process. These forces may give rise to different behavior when treated separately or when treated together simultaneously [21,22]. The prepared ECG skin electrodes underwent up to 50 wash cycles. Surface resistance was measured using a four-probe device, and the Ri/Ro (ratio of change in surface resistance from the original value) was calculated. ECG measurements were taken before and after washing. Six samples for each type of fabric were developed using both the silk and express wash cycles.

**Figure 3:**
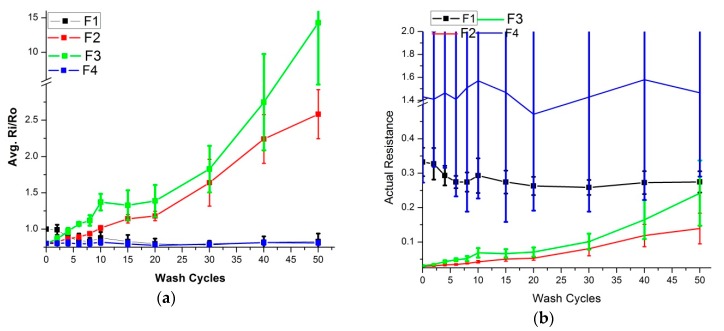
Surface resistance analysis (silk wash). (**a**) Comparison of Ri/Ro (**b**) Comparison of actual surface resistance.

Figure 3 shows the surface resistance behavior in the response of silk wash cycles for the fabricated skin electrodes (F1–F4). Avg. Ri/Ro is the average resistance change for all samples of that type (F1–F4, E1–E2). For comparison, actual resistance (Ohm/Sq.) is also plotted on separate graphs. The surface resistance properties of the F1 and F4 skin electrodes did not change, even after 50 wash cycles. They were both silver-based fabric electrodes, and their initial surface resistance was higher than those of F2 and F3. The initial resistance of F1 and F4 was 0.33 and 1.45 ohm/sq., whereas it was 0.038 and 0.042 ohm/sq. for F2 and F3, respectively. Both F2 and F3 were copper-based electrodes, and resistance started increasing after the first wash cycle. After 50 wash cycles, resistance had increased 10 and 15 times respectively for the F2 and F3 type electrodes. Their final surface resistance was 0.35 and 0.65 ohm/sq. respectively. This was less than the F1 and F4 surface resistance, even after the 15-times increase compared to the original values. These values were adequate to get ECG signals of good quality, thanks to the very low initial values. In the case of silk wash, all four types of electrodes gave acceptable ECG signals, even after 50 wash cycles.

**Figure 4:**
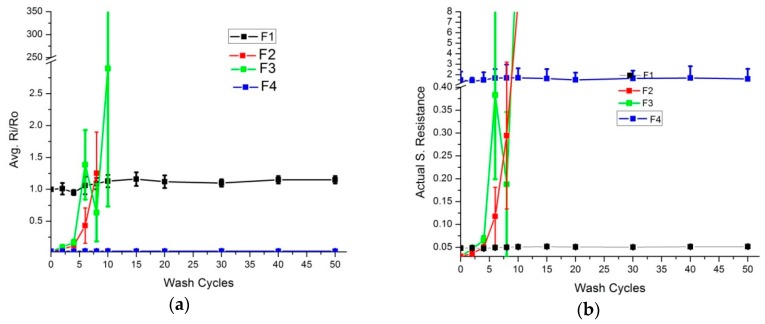
Surface resistance analysis (express wash). (**a**) Comparison of Ri/Ro (**b**) Comparison of actual resistance.

Express wash cycles, as explained earlier, have higher washing speeds, and more intense mechanical actions occur during the wash. In this case (Figure 4), the F2 and F3 electrodes completely lost their conductivity after 10 wash cycles. The F1 and F4 skin electrodes retained their conductive behavior, and resistivity increase was less than 1.15 times the initial values.

**Figure 5:**
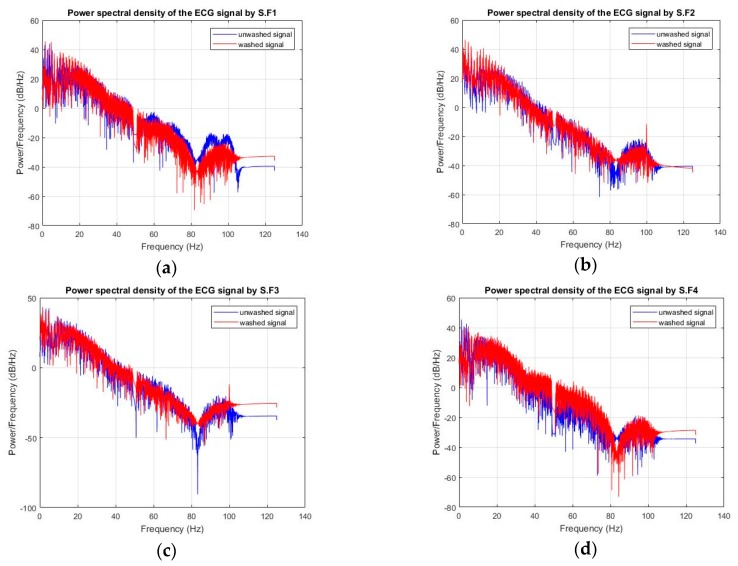
Power spectrum density (Silk wash), (**a**) F1 (**b**) F2 (**c**) F3 (**d**) F4.

**Figure 6:**
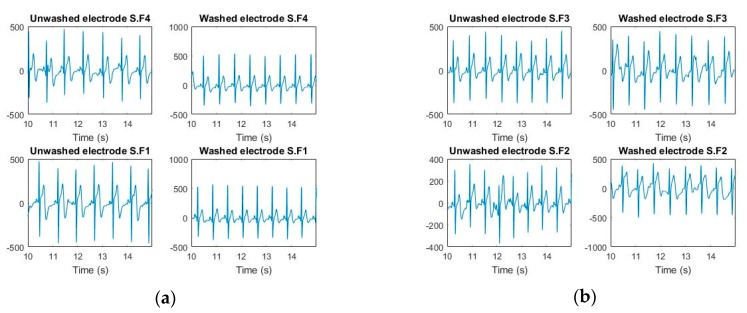
ECGs measured (silk wash). (**a**) F1 and F4 (**b**) F2 and F3.

**Figure 7:**
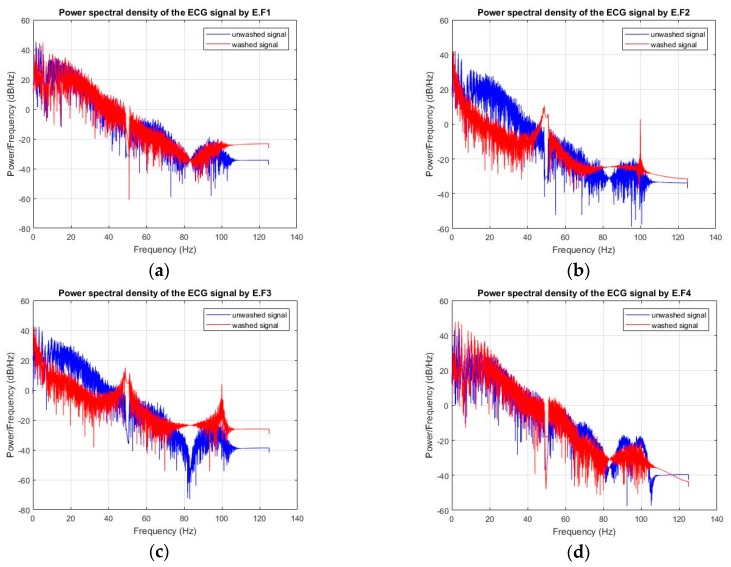
Power spectrum density (express wash), (**a**) F1 (**b**) F2 (**c**) F3 (**d**) F4.

**Figure 8:**
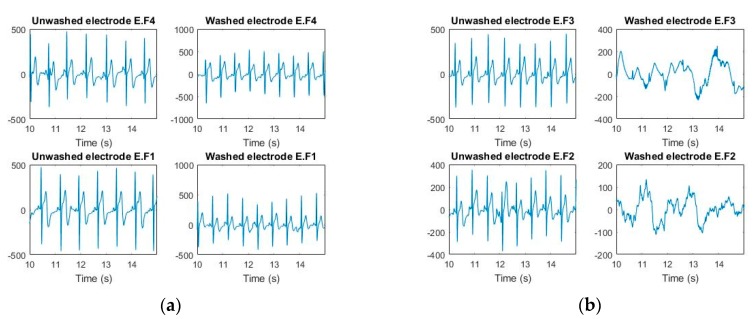
ECGs measured (express wash). (**a**) F1 and F4 (**b**) F2 and F3.

ECG monitoring was conducted before and after 50 wash cycles for all types of analysis. For the silk wash process, all samples detected the electrocardiographic waves clearly after 50 wash cycles (Figure 5 and Figure 6). The P, Q, R, S, and T waves and QRS complex can be easily separated in these curves. Power Spectrum Density (PSD) before and after washing was plotted for all samples. At frequency domain < 5Hz (ECG signal domain), the power spectrum density was not degraded for any of the samples. For express wash cycles (Figure 7 and Figure 8), the F2 and F3 electrodes did not yield any signal, and P, Q, R, and S waves were not detectable. The power spectrum density for these electrodes also showed degradation all over the spectrum, and even at less than 5Hz for these electrodes.

**Figure 9:**
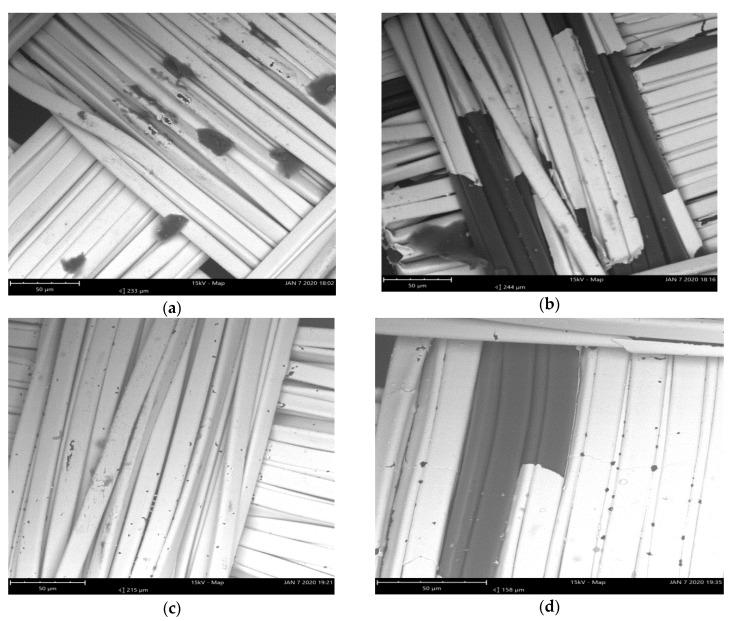
SEM analysis, (**a**) F3 before wash (**b**) F3 after wash (**c**) F2 before wash (**d**) F2 after wash.

In SEM (Scanning Electron Microscope) analysis, as shown in Figure 9, it was observed that copper layers were peeled off from base polyester fibers after washing. On the other hand, silver-based electrodes were not damaged, and only minor holes in some places were observed. The adhesion between the copper/nickel and base nylon fiber was not good enough to withstand the wash process, and it started peeling off after a few wash cycles. Copper has very good conductive properties that were visible from the initial very low surface resistance values compared to the silver base electrodes. However, resistivity increased rapidly after the wash cycles. This phenomenon occurred more quickly in the express wash because of the higher mechanical forces acting on the electrodes. So, we can conclude that copper/nylon adhesion can only bear mild wash cycles. Silver-based electrodes have very good adhesion with base nylon fibers and could withstand both silk and express wash processes without increasing the surface resistance.

**Figure 10:**
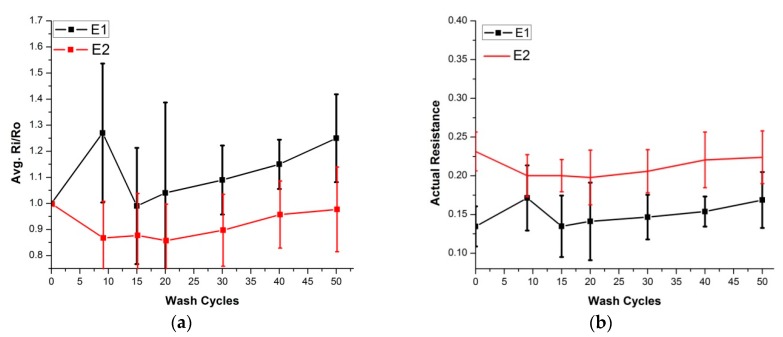
Surface resistance analysis (silk wash), four samples of each electrode were tested, (**a**) Comparison of Ri/Ro (**b**) Comparison of actual resistance.

**Figure 11:**
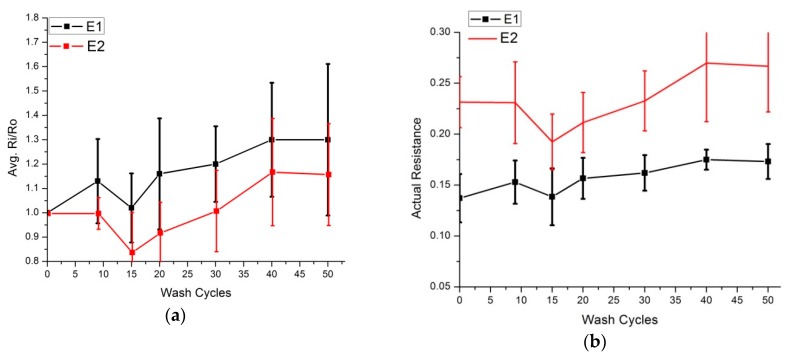
Surface resistance analysis (express wash), four samples of each electrode were tested, (**a**) Comparison of Ri/Ro (**b**) Comparison of actual resistance.

The same practice was repeated for embroidered skin electrodes prepared for ECG analysis. The E1 and E2 electrodes had an initial surface resistance of 0.14 and 0.23 ohm/sq. respectively. They underwent up to 50 wash cycles in both silk and express wash cycles. In both wash modes, the surface resistance increase was nominal (Figure 10 and Figure 11), i.e., in all samples, it was less than 1.3 times the initial value.

**Figure 12:**
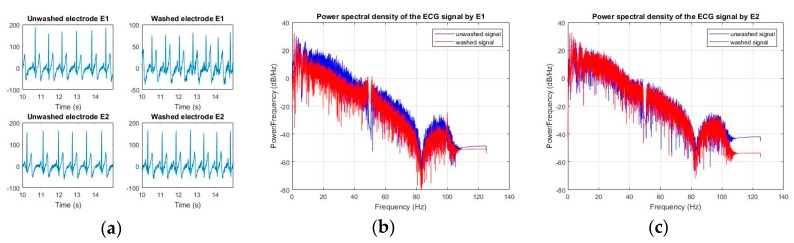
ECG analysis (express wash), (**a**) ECG signals for E1 and E2 (**b**) power spectrum density E1 (**c**) power spectrum density E2.

After 50 wash cycles, for both E1 and E2 electrodes, the electrocardiographic waves were able to detect all peaks, and the power spectrum density did not degrade at a low frequency suitable for ECG (Figure 12). In a comparison with E1 and E2, surface resistance change in E1 was greater than that of E2; this change was also visible in the power spectrum density. For E1, we can see small degradation in the low frequencies after 50 wash cycles.

### 3.2. Abrasion Resistance

**Figure 13:**
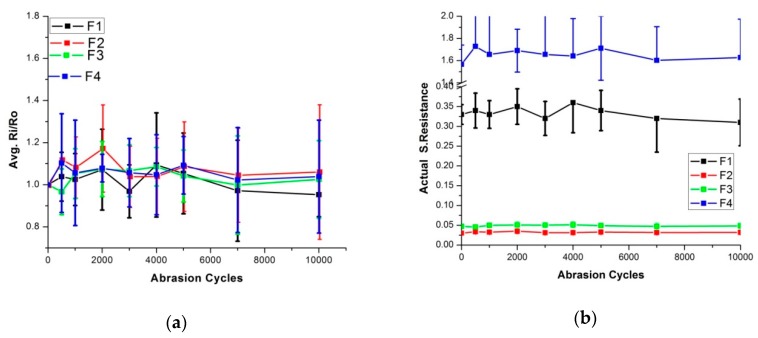
Surface resistance analysis after abrasion cycles, (**a**) Comparison of Ri/Ro (**b**) comparison of actual resistance.

Wash cycles exert different types of forces acting together during the washing process. Chief among them are mechanical forces, as explained previously [8]. To simulate these mechanical forces, Martindale abrasion resistance experiments were executed. However, the effect of mechanical forces performed separately may be changed from the effect when performed in combination with other forces during the wash process. But these tests were performed initially to make a rough assessment of damages. A total of 10,000 abrasion cycles were performed on all types of skin electrodes. Figure 13 explain the change of surface resistance in relation to abrasion cycles for F1 to F4 type of skin electrodes. In all these electrodes, the change of surface resistance was minimal after 10,000 abrasion cycles. 

In comparison with the wash analysis, results can be justified for F1 and F4 electrodes, because they also did not change their characteristics after 50 wash cycles. However, F2 and F3 gave unexpected results. These electrodes were damaged in the wash process, but showed stable performance in abrasion cycle experiments. These electrodes were heavily damaged after 10 wash cycles in the express wash, but they were stable in silk wash cycles and gave acceptable values after 50 silk wash cycles. The silk wash cycle demonstrated less mechanical damage compared to the express wash, and these samples were good enough to withstand these forces. So, the hypothesis can be put forward that abrasion resistance forces were not enough to peel off the copper layer from the base nylon material. As explained, these forces may work differently when acting together. There is a possibility that this peeling process was enhanced when mechanical forces acted together with chemical forces. To further investigate these concepts, chemical forces were also investigated separately.

**Figure 14:**
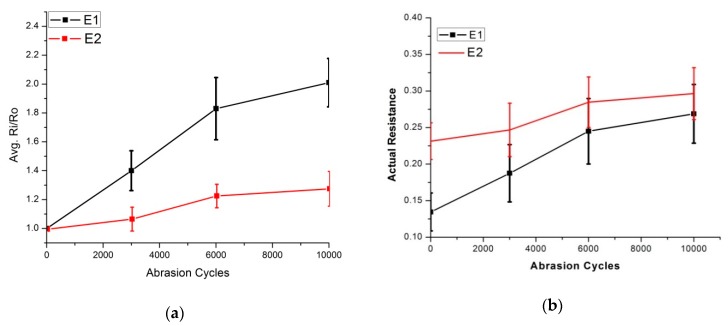
Surface resistance analysis after abrasion cycles, four samples of each electrode were tested, (**a**) Comparison of Ri/Ro (**b**) comparison of actual resistance.

Embroidered skin electrodes were also investigated over up to 10,000 abrasion cycles, and changes in surface resistance were recorded (Figure 14). E1 electrodes increased their surface resistance almost 2 times; in the case of E2, it was 1.2 times. Surface resistance after 10,000 abrasion cycles was 0.26 ohm/sq. and 0.29 ohm/sq. for E1 and E2 respectively. We observe in the wash process that E1 electrodes showed more damage compared to E2 electrodes. The same trend is also visible in Martindale abrasion testing. In this case, we can conclude by abrasion testing that E1 is more fragile to mechanical actions than E2. These predictions can be established commercially and can be used for product verifications without actually washing the prototypes.

### 3.3. Pilling Resistance

**Figure 15:**
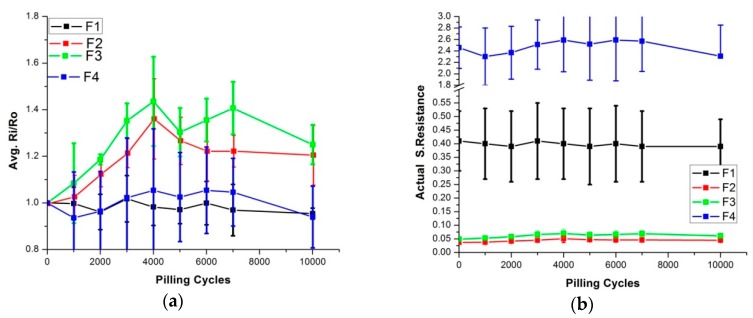
Surface resistance analysis after pilling cycles. (**a**) Comparison of Ri/Ro (**b**) comparison of actual resistance.

Another available mechanical test method that can be assumed to produce similar damage as to the wash process is the pilling box test. In the wash process, samples were revolved in a wash drum. The same type of phenomenon occurs in the pilling box test. Samples were investigated over up to 10,000 pilling box cycles, assuming as they were rotated in wash drum during the washing process. Surface resistance was measured in relation to pilling cycles, and changes in resistance were calculated (Figure 15). The same trend as that shown in the washing measurement and Martindale abrasion measurement was also visible here. F2 and F3 showed about a 1.2-times increase in surface resistance after 10,000 abrasion cycles. Although this increase is negligible, it is still notable in comparison with F1 and F4. Here again, the hypothesis can be put forward that F2 and F3 underwent some damage after 10,000 cycles, and that they are more fragile to mechanical damage. On the other hand, F1 and F4 did not increase their surface resistance. So, we can conclude that in these experiments, copper-based skin electrodes were exposed to mechanical forces more easily compared to silver-based skin electrodes. Error bars for F1 and F4 showed higher standard deviations in all samples. As explained, these are knitted samples, and hence, are more flexible compared to the others. They also have small pores due to the knitting techniques used, which generates rough surfaces with loops and empty spaces between them. This increased variations in the surface resistance measurements, as shown by four probe measuring units for both knitted electrodes. It was concluded that the available mechanical test methods could be used to predict potential damages that may accumulated due to washing. These predictions may be different for different types of materials, and can be used as an indication for a target material.

### 3.4. Chemical Analysis

During the wash process, chemical forces are the second most impacting force after mechanical forces. These include both water and water detergent solutions acting on electrodes during the wash process. Skin electrodes were dipped in water and water detergent solutions separately for a maximum of 72 h. The temperature of both solutions was kept at 40 °C to avoid damage provoked by the difference in swelling at high temperatures (i.e., above 75 °C) between the core polymer and the plated metallic layer.

**Figure 16:**
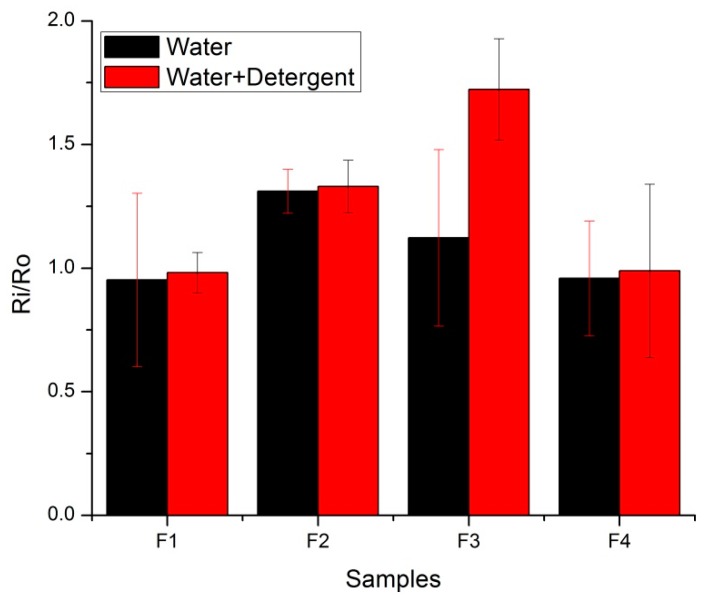
Surface resistance analysis with immersion in solutions (F1–F4 electrodes).

Surface resistance was measured after each 30 min, 2 h, 12 h, 24 h, 48 h, and 72 h. Figure 16 illustrates the changes in resistance after 72 h for all the fabricated skin electrodes. As changes in resistance for initial time frame were negligible, only the final time duration is plotted. The same trend was observed in this analysis. The F1 and F4 electrodes did not change their surface resistance after 72 h in the water and detergent solution. The surface resistance for F2 and F3 increased in both water and the water detergent solutions. In the water detergent solution, resistance was increased 1.3 and 1.7 times for F2 and F3, respectively. Copper-coated fabric electrodes were chemically attacked, and resistance increased without any mechanical force acting upon it. 

Water molecules alone or with detergent particles can chemically attack the surface of the electrodes or oxidize them. Oxygen present in water can corrode copper molecules; this phenomenon increases in intensity at high temperatures [23,24]. Broo et al. [25] discussed copper corrosion after dipping specimens in water for a calculated period. They claimed that with increasing time, cuprous oxide (Cu_2_O) density was increased. This corrosion can reduce the adhesion bonding between metal and inner nylon materials. This phenomenon can be further damaging together when combined with mechanical forces.

**Figure 17:**
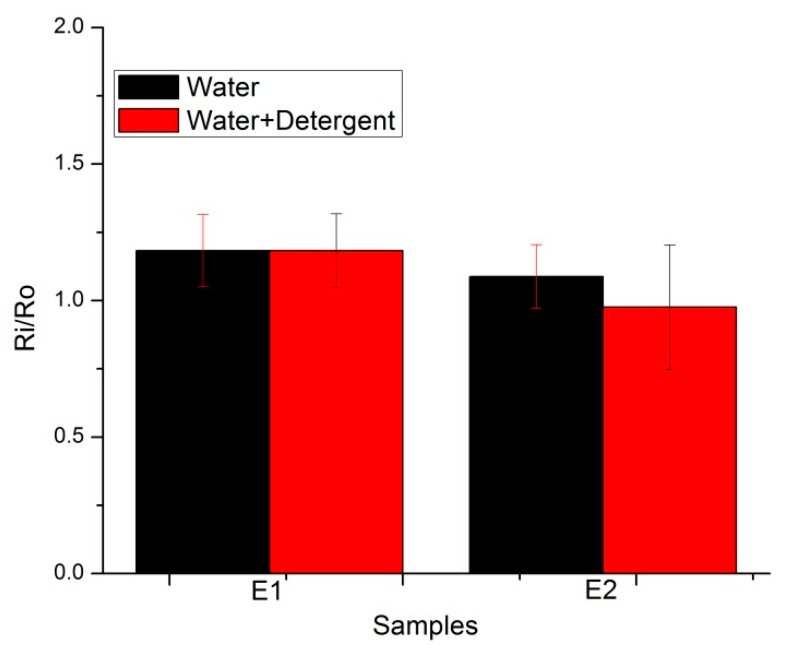
Surface resistance analysis with immersion in solutions (E1–E2 electrodes).

The same experiment was repeated for embroidered skin electrodes. ECG electrodes were dipped in water and water detergent solutions for different times, ranging 30 min, 2 h, 12 h, 24 h, 48 h, and 72 h. Here again, only the final (72 h) results are plotted. Figure 17 explains the changes in resistance after 72 h’ dipping in water and water detergent solutions. The same trend was visible when we compared E1 and E2. In the wash process, E2 showed better resistance to degradation compared to E1, a notable but small and negligible trend.

After these exercises, we can conclude that copper-coated skin electrodes were readily degraded through the chemical effects of water and water detergent solutions, reducing the adhesion between the base nylon and copper coating. This weak adhesion further suffered and started tearing off in the presence of mechanical forces acting upon it. As the intensity of these forces increased, e.g., in the case of express washing, the skin electrodes are heavily damaged.

## 4. Discussion

This article highlights the impact of different wash forces on e-textiles. Skin electrodes using a variety of materials were prepared and underwent up to 50 wash cycles in silk and express mode wash processes. Different damage generating factors were investigated and their impact on performance reliability was discussed. Some available textile testing methods are suggested to predict the wash damage of electrodes without actually washing them.

Four fabricated and two embroidered skin electrodes were investigated in these experiments. In the fabricated electrodes, two types of copper-coated and two silver-coated skin electrodes were examined. Silver-coated electrodes showed stable performance after 50 wash cycles in both wash modes. Copper-coated electrodes increased their resistance with increasing wash cycles. In silk wash mode, resistance increased up to 15 times, but this was still low enough to get good ECG signals, as copper has very good conductive properties and these electrodes had an initial resistance that was about 10 times less than that of the silver-based skin electrodes. So, even after the a 15-fold increase in resistance, it was good enough to get an ECG signal. In express wash mode, copper-based electrodes were damaged after 10 wash cycles, and we could no longer get an ECG signals. Express wash modes have harsher mechanical forces compared to silk wash mode. We can therefore state that these electrodes are able to withstand certain levels of damaging forces, beyond which their performance will degrade. It is predicted that the adhesion between copper and base nylon fiber was not good enough, and the copper layer started peeling off after the wash process. The express wash mode accelerated this process. SEM analysis further confirmed this.

Martindale abrasion tests and pilling box tests were performed on the textile dry skin electrodes. These mechanical tests also confirmed the hypothesis. Copper-based electrodes increased their resistance after 10,000 abrasion and pilling box cycles. Although this increase was not as marked as that of the wash process, it was still visible in comparison to the silver-based electrodes, and can be used to predict the behavior of different types of electrodes being subjected to washing. 

To further investigate wash damage, these electrodes were dipped in water and water detergent solutions at 40 °C for 72 h. Here again, a similar trend was observed. Silver-based electrodes showed no change in resistance, but copper-based electrodes increased their resistance.

Our previous work [8,26] explained different types of wash forces that can play different roles in the wash process. In these experiments, these forces were further investigated on different skin electrodes, and possible alternative mechanical and chemical tests are suggested. These test methods can be used to predict the washability of specific types of e-textile components without actually subjecting them to washing. These results may be helpful in developing standards in the future. In future experiments, these results will be validated on different e-textile components to develop washability standards for e-textiles.

## Figures and Tables

**Table 1:** Sample coding.

Sample #.	Material	Brand
E1	Silver coated Yarn- Embroidered	Shieldex- 117f17 HC+B
E2	Silver coated Yarn- Embroidered	Madeira HC-40
F1	RF Shielding Silver Fabric	Faradaydefense LLC
F2	RF Shielding Nickel Copper Fabric	Faradaydefense LLC
F3	RF Shielding Copper Fabric	Faradaydefense LLC
F4	Silver-plated Fabric	Innovative textile
